# Freeze-Drying of Plant-Based Foods

**DOI:** 10.3390/foods9010087

**Published:** 2020-01-13

**Authors:** Sagar Bhatta, Tatjana Stevanovic Janezic, Cristina Ratti

**Affiliations:** 1 Département Sciences du Bois et de la Forêt, Institute of Nutrition and Functional Foods, Université Laval, Quebec City, QC G1V 0A6, Canada; sagar.bhatta.1@ulaval.ca (S.B.); Tatjana.Stevanovic@sbf.ulaval.ca (T.S.J.); 2 Département des Sols et de Génie Agroalimentaire, Institute of Nutrition and Functional Foods, Université Laval, Quebec City, QC G1V 0A6, Canada

**Keywords:** freeze-drying, lyophilization, plant-based foods, fruits, vegetables

## Abstract

Vacuum freeze-drying of biological materials is one of the best methods of water removal, with final products of highest quality. The solid state of water during freeze-drying protects the primary structure and the shape of the products with minimal volume reduction. In addition, the lower temperatures in the process allow maximal nutrient and bioactive compound retention. This technique has been successfully applied to diverse biological materials, such as meats, coffee, juices, dairy products, cells, and bacteria, and is standard practice for penicillin, hormones, blood plasma, vitamin preparations, etc. Despite its many advantages, having four to ten times more energy requirements than regular hot air drying, freeze-drying has always been recognized as the most expensive process for manufacturing a dehydrated product. The application of the freeze-drying process to plant-based foods has been traditionally dedicated to the production of space shuttle goods, military or extreme-sport foodstuffs, and specialty foods such as coffee or spices. Recently, the market for ‘natural’ and ‘organic’ products is, however, strongly growing as well as the consumer’s demand for foods with minimal processing and high quality. From this perspective, the market for freeze-dried plant-based foods is not only increasing but also diversifying. Freeze-dried fruits and vegetables chunks, pieces, or slices are nowadays majorly used in a wide range of food products such as confectionaries, morning cereals, soups, bakeries, meal boxes, etc. Instant drinks are prepared out of freeze-dried tea, coffee, or even from maple syrup enriched with polyphenol concentrated extracts from trees. The possibilities are endless. In this review, the application of freeze-drying to transform plant-based foods was analyzed, based on the recent research publications on the subject and personal unpublished data. The review is structured around the following related topics: latest applications of freeze-drying to plant-based foods, specific technological problems that could be found when freeze-drying such products (i.e., presence of cuticle; high sugar or lipid concentration), pretreatments and intensification technologies employed in freeze-drying of plant-based foods, and quality issues of these freeze-dried products.

## 1. Introduction

Plant-based foods, including fruits, vegetables, seeds, beans, spices, etc., are important components of a healthy diet, and their sufficient regular consumption could help to prevent certain major diseases such as cancer and cardiovascular diseases, etc. According to the combined report of World Health Organization and Food and Agriculture Organization, it was recommended that a daily minimum consumption of 400 g of fruits and vegetables may help to minimize the occurrence of chronic diseases along with the mitigation of micronutrient deficiencies [1]. Fresh plant-based foods may not available all year round for consumption and the long-term storage of fresh foods could be challenging due to high water content, unavailability of cold-storage facilities (particularly in underdeveloped and developing countries), and possibility of nutritional deterioration. Consequently, drying of such foods may allow their long-term consumption and eases handling, transportation, and storage.

Freeze drying (FD), also known as lyophilization, is a well-known technique for the production of high quality food powders and solids [2,3]. It is a preferred method for drying foods containing compounds that are thermally sensitive and prone to oxidation since it operates at low temperatures and under high vacuum. Application of FD to various plant-based foods, such as apple, guava, strawberry, blackberry, pumpkin, tomato, asparagus, coffee, tea, garlic, ginger, maple syrup, etc., has already been reported in literature [4,5,6,7,8,9,10,11,12,13,14,15].

Drying of food results in food quality changes [3]. The quality of foods can be divided into three sections: physical, chemical, and nutritional. Major qualities of foods that can be affected during drying processes are color, odor, texture, rehydration property, bulk properties, flow property, water activity, and retention of nutrients and volatile compounds [16]. Regarding to nutritional qualities, oxygen, high temperature, and cell damage are usual enemies of bioactive compound retention during processing. The stability of the valuable compounds of plant-based foods can therefore be affected during dehydration. Phenolic compounds could be susceptible to enzymatic degradation due to the polyphenol oxidase activity [17]. In addition, carotenoids have a highly unsaturated nature, making them susceptible to degradation by oxidation and thermal processes. Oxidation is the major cause of carotenoids degradation and can be generally considered autocatalytic, beginning only after an induction period in which radicals are built up and antioxidants are depleted [18]. Also, the loss of vitamin C and carotenoids is affected particularly by the temperature and the moisture content during drying processes [19]. Vitamin C is usually selected as a reference index of the nutrient quality due to its labile nature compared to other nutrients in foods [20], and thus, if ascorbic acid is well retained, other nutrients would be as well. A few interesting reviews on the impact of drying methods and operating conditions on functional quality retention can be found in the literature [16,21].

Freeze-drying method by lack of liquid water, oxygen-free environment (if operated under vacuum), and low operating temperatures is thus the best choice to dehydrate fruits and vegetables in order to keep an optimized biocompound content in the final products. Despite the long drying time and expensive process, freeze-drying is widely used to produce high-value food products due to maximal retention of food quality when compared to other drying techniques. FD is considered as the standard or reference drying method in most research studies. Lately, various process intensification approaches have been implemented in order to overcome the challenges facing by FD methods, such as either pretreatment of the sample or the use of innovative technologies including infrared, microwave, and ultrasound energy with freeze-drying.

The aim of current review was to highlight the application of FD of plant-based foods, to point out some particular technological challenges, and describe process intensification in FD of plant-based foods to improve the quality of freeze-dried foods or to accelerate the process.

## 2. Methodology

The following databases were used for a bibliographic research: Web of Science (2000–2019) and Google Scholar (2000–2019). Authors have presented some of their original works (experimental data and images) to support conclusions.

## 3. FD Principle

**Figure 1:**
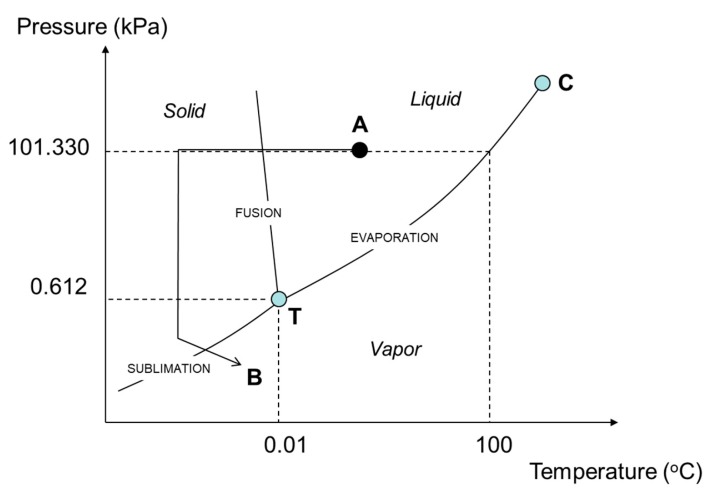
Phase diagram of water (T: triple point of water, C: critical point of water). “A” represents the starting point prior to freeze-drying (atmospheric pressure and ambient temperature), while “B”, the desired final conditions during sublimation (below the triple point T).

Water exists in three different states: solid, liquid, or gas (vapor). Figure 1 presents the phase diagram of water (pressure versus temperature), where the curve lines show the passage from solid to vapor (sublimation), from liquid to vapor (evaporation), or from solid to liquid (fusion). Point T in Figure 1 represents the triple point of water (at 0.01 °C and 0.612 kPa) where the three phases (liquid, vapor, solid) coexist, and point C is the critical point of water (374 °C and 22060 kPa). Freeze-drying makes use of the sublimation phenomenon (at temperatures lower than 0.01 °C, and water vapor pressures below 0.612 kPa). In Figure 1, a product to be freeze-dried will follow the path from A to point B (i.e., the product should be first frozen by decreasing its temperature, then the water vapor pressure should be lowered below the pressure corresponding to the triple point, and finally some heat should be supplied to help the ice to convert into vapor by sublimation).

During the FD process, the removal of solid-state water (ice) occurs in three steps: (a) freezing, where the sample should be completely frozen; (b) primary drying, when ice is sublimated, usually at sub-atmospheric pressure; and (c) secondary drying, when the remaining unfrozen/bound water is desorbed from the drier food matrix.

Freezing is the first separation step in the FD process, which solidifies the food materials. The rate of freezing is important for the formation and size of ice crystals—slow rate of freezing forming bigger ice crystals and vice versa. Accordingly, the size of crystals affects the rate of drying, wherein large ice crystals are easier to sublimate and hence increase the rate of primary drying [22]. 

**Figure 2:**
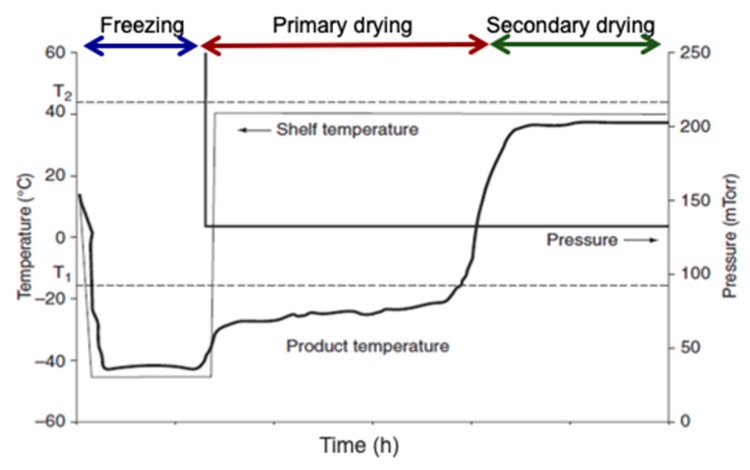
Temperature profile of product during freeze-drying process, where T_1_ (dotted line) is the collapse temperature and T_2_ (dotted line) is the glass transition temperature of dry solids (adapted from [22]).

In primary drying, a vacuum is applied and the shelf temperature is increased to start the sublimation, such that the product temperature is 2–3 °C below the collapse temperature *T_c_* [23,24]. Collapse temperature is the temperature above which the product has the risk of losing macroscopic structure during the FD process [24]. *T_c_* could be determined with a freeze-drying microscope, but also may be estimated from the glass transition temperature (*Tg*). It should be noted that *Tc* could be 2 °C to 20 °C higher than *Tg*, depending mainly on sample composition [24,25]. However, very conservative predictions of the collapse temperature may only result in a much longer freeze-drying process, thus it can only be used in critical cases when the sample is difficult to freeze-dry. Figure 2 depicts the typical temperature profile of a product during each step of the freeze-drying process, where it can be observed that during primary drying the product temperature should be below the collapse temperature (represented as a dotted line T_1_ in Figure 2).

Secondary drying starts when sublimation is still in place, being a slow part of the freeze-drying process, which may take at least 30% longer to complete than the end of sublimation. This last step could be performed at an elevated shelf temperature to more efficiently remove the remaining unfrozen or bound water by desorption, but lower than the glass transition temperature of dry solids (represented as dotted line T_2_ in Figure 2). However, it is challenging to identify the endpoint of primary drying or the beginning of secondary drying phase. If temperature is increased before all the ice is sublimated (endpoint of primary drying phase), it could collapse the product and hence, affect the final quality. Patel et al. [23] have suggested some techniques to determine the endpoint of the primary drying such as Pirani pressure gauge, dew point monitor, tunable diode laser absorption spectroscopy (TDLAS), gas plasma spectroscopy, thermocouple (TC), and condenser pressure. Among these techniques, Pirani, dew point, TDLAS, and TC were found to be effective for determining the endpoint of the primary drying phase.

## 4. Characteristics of Plant-Based Foods, Their Advantages, and Challenges upon Freeze-Drying

Plant-based foods are derived from vegetables, grains, nuts, seeds, legumes, and fruits [26]. Two types of plant-based foods are used in freeze-drying applications: solids and homogeneous solutions/suspensions such as juices or purees.

**Figure 3:**
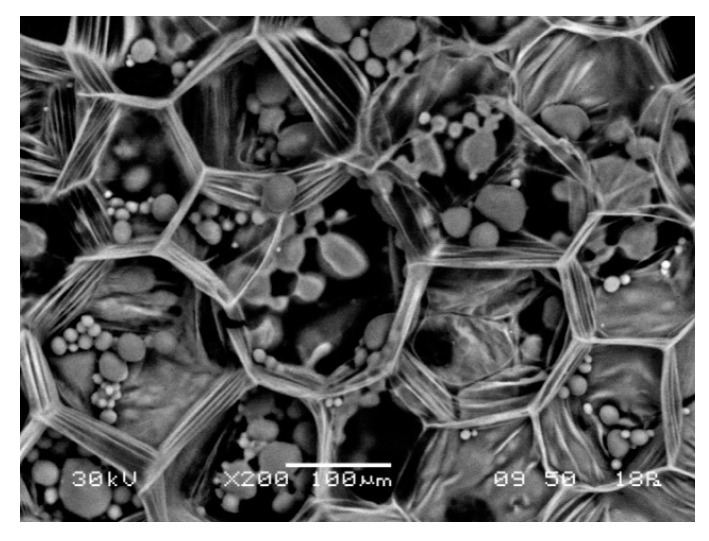
Cellular structure of potato.

Solid plant foods present intrinsic characteristics in terms of structure, anatomy, and composition, which may pose challenges on one hand, but otherwise occasionally help to ease the freeze-drying operation. To start, solid plant-based foods are mainly cellular solids. Gibson [27] reported that apples and potatoes are examples of a simple cellular tissue: parenchyma with thin-walled, polyhedral cells resembling an engineering closed-cell foam, as shown in Figure 3 for potato [28]. Unlike solutions and colloidal systems, cellular solids present stronger mechanical attributes related to the properties of the cell wall material and to the cell geometry—cellular materials allowing the simultaneous optimization of stiffness, strength, and overall weight in a given application [29]. Cellulose and noncellulosic (hemi-celluloses and pectic) polysaccharides are the main polymers forming the cell wall of plant-based foods. Cellulose is the single most abundant polysaccharide component of vegetable cell walls, presenting areas of crystallinity imparting a considerable tensile strength close to 1 GPa, and with a Young’s modulus roughly 130 GPa [27,30]. Also, the mechanical response of cellular materials is enhanced by their arrangement and local geometry [31]. In this sense, if the freezing step is properly done at adequate low temperatures (without ice crystals destroying/weakening the cell walls), cellular materials are better prepared to stand during freeze-drying. It can be said that when freeze-drying solid cellular foods, mechanical properties and structural strength may play a more important role in keeping product integrity than glass transition temperature in order to avoid collapse during primary/secondary drying and storage of freeze-dried foods.

**Figure 4:**
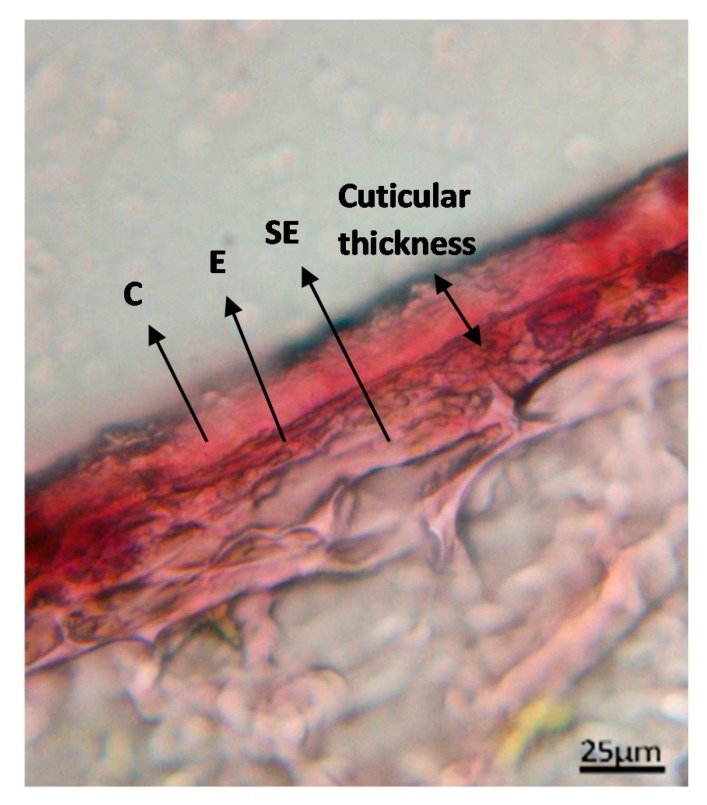
Optical microscope photo of a blueberry epidermis zone, where *C* is the proper cuticle, *E* is the epidermis, and *SE* is the sub epidermis.

Most plants present an epidermis in their outer parts serving against water loss, regulating gas exchange, and secreting metabolic compounds to protect internal tissues against diseases and acting as a natural insect repellent as well. Figure 4 shows the cross section of the epidermal cuticle of *Vaccinium angustifolium* (lowbush) blueberries (magnified 250 times) [32]. This epidermis formed by a lipidic hydrophobic cuticle layer [33] constitutes an interface between the internal cells and the external environment, acting as a moisture barrier during growing, which enormously affects the water diffusion during subsequent processing, decreasing significantly the rate of freeze-drying when the whole plant-based material is dried (i.e., the case of berries/grapes). The outer surface of the cuticle is covered by epicuticular waxes (a lipid-soluble fraction) and consists of complex mixtures of long-chain aliphatic and cyclic components, including primary alcohols (C26, C28, C30), hydrocarbons (C29, C31), esters, fatty acids, and triterpenoids [34,35]. Intracuticular waxes are embedded in the cutin polymer matrix itself (a lipid-insoluble fraction), though little information is available on its composition [36]. This external waxy layer makes freeze-drying of whole fruits/vegetables challenging since vapor generated by ice sublimation during the primary step is trapped inside the product, increasing its pressure and thus, melting the ice. Finally, after a continuous pressure build-up, the product cracks or explodes inside the freeze-dryer, depending on the vacuum level. The quality of such freeze-dried product is therefore unacceptable, and thus, pretreatments are required to overcome this problem (please refer to Section 6).

For plant-based liquid solutions, sample composition may affect freeze-drying operation depending on one hand by the type of compound and its impact on overall glass transition temperature, but also on the total concentration of solids.

In a glass state, the viscosity of the matrix is high and the molecular movement is very limited. Glass transition occurs when a glassy matrix changes to a rubbery state, which is a more mobile amorphous structure. As explained previously, collapse temperature (related to the glass transition temperature *Tg*) represents the temperature above which the matrix loses its structure and the quality decreases, obviously related to the decrease in viscosity happening during glass transition. Therefore, when temperature during a process increases over the *Tg* of a product, the deterioration risk of many of its physical properties rises as well. Table 1 shows an example of the carbohydrate composition of apple and pear [37] together with literature values of the glass transition temperatures of pure sucrose, glucose, fructose, and D-sorbitol. As can be seen, pear juice has higher mass fractions of glucose and sorbitol than apple juice, and lower fractions of sucrose and fructose. Glass transition temperature of a multicomponent mixture could be roughly estimated using the following equation:

(1) $$Tg=\overset{n}{\underset{i=1}{\sum }}x_{i}Tg_{i}$$

where *x_i_* and *Tg_i_* are the mass fraction and individual glass transition temperature of each component, respectively. Water is a plasticizer, having a low glass transition temperature, and some authors have indicated a value of −137 °C [38]. Thus, while drying takes place, the glass transition temperature of a product increases as water content is reduced, as indicated in Equation (1). Equation (1) usually underestimates experimental glass transition values [25], however, it can be used in this manuscript to predict the effect of composition for comparison purposes. From this equation, and using the mass fractions of Table 1, the predictions of glass transition temperature for dry pear juice (≈ 60.9 °C) are approximately 16 degrees lower than for apple juice (≈ 77.1 °C), probably due to its lower mass fraction of fructose and higher mass fraction of sorbitol. Experimental values for dried apple and pear showed a similar difference in glass transition temperatures values of an average of 10 degrees [39], thus validating the general conclusions obtained from Equation (1).

From the previous discussion, it can be said that pear juice would have less thermal stability than apple juice upon freeze-drying at similar operating conditions, provoking final freeze-dried pear juice with lower quality (i.e., darker, stickier, lower rehydration, etc.). These predictive results have been corroborated by experimental freeze-drying data [37]. In these cases, the product with lower glass transition has to be freeze-dried at lower shelf temperatures and under higher vacuum, making the process longer and increasing costs. This example aimed to illustrate the utmost importance that composition, and its influence in glass transition, has for freeze-drying of liquid plant-based foods, such as juices. As indicated in [41], the collapse temperature of pure orange juice is relatively low, the dry juice collapsing at 52 °C. This collapse temperature is very close to that of sucrose (55 °C), due to the higher sucrose content of this juice (more than 50% of the sugars). In the same study, it was shown that addition of macromolecules increases the collapse temperature of freeze-dried orange juice, thus providing better thermal stability.

In cellular solid foods, collapse during drying takes place when the natural turgidity of product is lost and cannot be restored. The impact of glass transition in this case is less important since structure plays a major role in understanding the collapse phenomena. In a freeze-drying study of potato, celery, and apple at temperatures below, near, and above their *Tg*, [42] pointed out that differences in plant tissues (structure, composition) may contribute together with glass transition to prevent collapse.

In particular for liquids (solutions, emulsions, suspensions), the matrix of the product to be freeze-dried provides for ‘body’, mechanical strength, and an attractive appearance [43]. Concentration of simple constituents of this matrix could thus have a significant impact on the freeze-drying operation since high levels of sugars or lipids may convert to low quality final freeze-dried products. For instance, when the sugar content is too high (i.e., concentrated orange juice, maple syrup), freezing temperatures should be set up at lower optimized levels for a successful freezing step. However, even if the temperature of the freezing step has been reduced, sugars might migrate to the surface of the product during freezing, building up a barrier for water diffusion that will affect the further primary drying step, having a similar role as the epidermis of berries mentioned earlier. Thus, as water has difficulty in escaping from the matrix, pressure builds up and the surface may crack, which is a positive solution for letting water to escape, but ice might melt, with goods exploding inside the freeze-dryer, producing final products with undesirable characteristics. Pretreatments should be used in such cases, as explained in Section 6.

Spray-drying is a continuous process considered industrially viable to produce powders out of fluids due to its lower costs compared to freeze-drying. However, when dealing with plant-based foods prone to collapse or containing valuable oxidative compounds, spray-drying at high temperatures and using enormous amounts of air is not as effective as a freeze-drying. Expensive complicated formulations are required to be added to solutions to be spray-dried in order to avoid quality problems related to oxidation, or important yield losses due to collapse and stickiness.

## 5. Application of FD to Plant-Based Foods

### 5.1. Fruits

Table 2 presents the wide range of freeze-dried fruits that have been reported in literature, including strawberry, blackberry, guava, pineapple, etc. [10,12,14,44,45,46,47,48]. Fresh fruits containing high moisture levels are difficult to dehydrate by classical drying techniques due to significant damage to their physical attributes, mainly collapse and severe bleeding due to the rupture of skin. Shishehgarha et al. [14] studied the various quality parameters (drying kinetics, color, and volume variation) of sliced and whole strawberries under different FD shelf temperatures (30, 40, 50, 60, and 70 °C). The level of shrinkage was found to be independent of FD shelf temperature, where whole and sliced strawberries had an average volume reduction of 8% and 2%, respectively. On the other hand, the risk of collapse was found to increase exponentially once the FD shelf temperature surpassed the glass transition temperature of dried strawberries [14]. Shrinkage and collapse of fruits are often encountered during drying, mainly by air-drying techniques. Hawlader et al. [12] compared quality of guava obtained by heat pump dryers (RH = 10%, v = 0.7 m/s, T = 45 °C, 8 h), vacuum (vacuum pressure = 15,000 Pa, T = 45 °C, 8 h), and freeze-dryers (freezing at −20 °C for 24 h, and less than 613.2 Pa vacuum pressure, 10 °C shelf temperature, 24 h for freeze-drying). Porosity, color, rehydration, and vitamin C retention of guava produced by FD were better, resulting in FD guava being the most desirable powder compared to that produced by vacuum and heat pump dryers. FD was able to retain 63% vitamin C, whereas heat pump dryer retained only 25%.

It is often challenging to dehydrate fruits with waxy impermeable skin, as described previously, such as seabuckthorn berries (*Hippophae rhamnoides* L.), which are delicate fruits containing natural antioxidants, ascorbic acids, carotenoids, and flavonoids. Araya-Farias et al. [44] investigated the effect of hot air drying (HAD) at 1 m/s and 50 °C or 60 °C, and FD (4 Pa of vacuum, 20 °C or 50 °C shelf temperature) to obtain dried seabuckthorn pulp (shown in Table 2). They found that FD retained 93% more carotenoids, 34% more vitamin C, and 11% more phenolics than HAD. Increased drying temperature resulted in increase of drying kinetics. However, no significant impact of drying temperature on overall nutritional retention was noticed for FD or HAD samples, although a slight decrease was found for particular compounds such as vitamin E for both drying methods while increasing the operation temperature. Between two drying processes, it was reported that drying kinetics were surprisingly faster in FD.

### 5.2. Vegetables

Vegetables are good source of essential nutrition for human diet. Dehydration of vegetables is usually done for their long-term consumption. Studies on FD of some vegetables, including asparagus, carrot, pumpkin, and tomato, are presented in Table 2. Nindo et al. [13] studied the drying of sliced asparagus using FD and other drying methods including tray dryer, refractance window dryer, and spouted bed dryer. The highest amount of ascorbic acid was retained when samples were dehydrated by FD and refractive window drying.

Freeze-dried pumpkin has numerous applications in manufacturing formulated foods such as soups, noodles, breads, and cakes. Several authors have studied the FD of pumpkin to characterize its nutritional and physicochemical properties for above-mentioned food applications [6,49,50]. Guiné et al. [49] reported the decrease in moisture content from 90% to 8% in freeze-dried pumpkin, but FD induced a softening of the pumpkin, as hardness of pumpkin decreased from 19.37 N (fresh) to 1.59 N (dried/rehydrated) when the textural properties were analyzed. Also, the change in color was important (total change in color, ΔE = 12). Ciurzyńska et al. [6] studied the effect of different pretreatment methods (blanching and osmotic dehydration) on the properties of freeze-dried pumpkin. The long duration of osmotic dehydration caused a decrease in water content of pumpkin and on water activity of final freeze-dried samples.

Gümüşay et al. [11] investigated the effects of sun, oven, vacuum-oven, and freeze-drying on the phenolic amount, antioxidant capacity, and ascorbic acid content of tomatoes. Freeze dried tomatoes had about twofold higher phenolic content than that of other drying methods (654.60 vs. 314.27–355.79 mg gallic acid equivalent/100 g dm). Unlike FD, processes using high drying temperatures may cause activation of oxidative enzymes, resulting in the loss of phenolic compounds. Enzymes such as peroxidative and hydrolytic may also have been liberated due to the disruption of tomato structure at high drying temperature [51]. The retention of ascorbic acid content (65.47 vs. 4.14–24.39 mg/100 g dm) and antioxidant capacity (1699.59 vs. 873.32–1148.86 mg trolox/100 g dm) was highest for freeze-dried tomatoes. Rajkumar et al. [52] reported high rehydration ratio and aroma retention in freeze-dried carrots. In terms of shrinkage, carrots exposed to FD had lower shrinkage rate (20.83%) than HAD (35.53%). Leafy vegetables such as spinach are also reported in the literature to be dehydrated by FD. An-Erl King et al. [53] found that the freeze-dried spinach had high porosity and surface area (263.6-296.8 m^2^/g). The chlorophyll content of freeze-dried spinach decreased with an increase in storage temperature and storage time.

### 5.3. Speciality Foods

The use of FD is not limited to fruits and vegetables; it has been used to produce dried specialty foods from plant sources such as coffee, tea, and spices [4,7,8,11,58,59,60,61,62,63,64,65].

Coffee and tea are the most popular beverages in the world. FD has been used to produce instant tea due to its ability to retain volatile compounds. Kraujalyte et al. [62] found that instant tea produced by FD had high concentration of volatile compounds (318.65 ng/g), which was comparatively two to five times higher than those produced by other drying methods (68.60 to 143.33 ng/g). In the case of coffee, the content of phenolic acids in coffee beans after FD was reported to increase by 41% more than in fresh green coffee beans [59]. A FD process for coffee has been recently designed using mathematical modeling to optimize energy efficiency and preserve the important flavors and nutrients [9]. Dong et al. [60] conducted a study to observe the effect of FD methods on odor compounds and the aromatic profile of roasted coffee beans. Interestingly, they found that quinic acid, which is a major organic acid that is attributed to coffee quality, was only detected in the sample dehydrated by FD method.

Spices such as garlic and ginger have also been reported to be dehydrated using FD. The effect of freeze-drying shelf temperatures on pore formation of garlic was studied by Sablani et al. [64]. They found that garlic dried at a higher shelf temperature resulted in lower open pore porosity. In addition, the apparent porosity of garlic exponentially increased with the drying time. Ratti et al. [63] investigated the effect of FD on allicin formation capacity. It was found that allicin content decreased with an increase in drying temperature and better retention of allicin formation capacity was obtained from the one dried at 20 °C. In another study, Fante & Noreña [8] found that FD garlic powder demonstrated better quality in terms of color and inulin content, and higher glass transition temperature when compared to forced HAD. High glass transition temperature of 44.9–46.2 °C in freeze-dried garlic powder can be related to low water activity of 0.12 to 0.13. Ginger, a common condiment used in a variety of foods and beverages, is another spice widely dehydrated using FD method [4,7]. FD of ginger led to high retention of gingerols, phenolic content, flavonoids, antioxidant activities, and some volatile compounds [4].

### 5.4. Nontraditional Source

More recently, there is has been an increasing trend of consumption of nontraditional foods or food from an alternative source. One such nontraditional plant source food is maple syrup. Maple syrup is composed of a mixture of sugars (66% sucrose, 0.4% glucose, and 0.5% fructose), minerals and water, and traces of organic acids, proteins, and polyphenols [66]. Bhatta et al. [67] studied the drying of maple syrup to produce a maple sugar powder. FD of sugar-rich foods is challenging due to high hygroscopicity of simple sugars, the increase in solubility with temperature, a low glass transition temperature of sugars (fructose, glucose, and sucrose; *Tg* = 5, 31, and 62 °C, respectively) [68], and the stickiness problem in the drying equipment [69]. The dilution of maple syrup from 66 to 20 °Brix was needed to produce a dried maple syrup powder. Such freeze-dried maple sugar powder exhibited an instant-like property as it dissolved within 14s, but showed fair to poor flow characteristics due to cohesiveness nature of sugars. Authors have also suggested the use of glass transition temperatures (related to collapse temperature) for the determination of FD temperatures and online temperature recording with thermocouples for the identification of drying periods [67].

### 5.5. Generalities about Impact of Freeze-Drying on Biocompounds

Several studies investigated/reviewed the important impact of different drying techniques, including freeze-drying, on the active ingredients and phytochemical contents of fruits, vegetables, and herbs and medicinal plants [70,71]. In particular due to freeze-drying, Marques et al. [57] showed significant losses in vitamin C in tropical fruits (3% to 70% depending on the fruit). Araya-Farias et al. [44] reported 20% loss in vitamin C and in total carotenoids, 35% loss in vitamin E, but only 4% loss in total phenolics when freeze-drying seabuckthorn berries at 20 °C shelf temperature and 30 mTorr vacuum pressure. So, although an excellent choice to preserve plant-based foods, freeze-drying cause some decrease in phytochemical content.

Nevertheless, when compared to other drying techniques, usually freeze-drying is a superior technology. Asami et al. [72] reported that freeze drying preserved total phenolics in marionberries, strawberries, and corn better than air drying. Sablani et al. [70] showed that compared to air drying, freeze drying improved retention of anthocyanins, phenolics, and antioxidant activity during processing of regular versus organic blueberries and raspberries, and in some cases it even increased the concentration of phytochemicals. Reyes et al. [73] also indicated that ascorbic acid content in blueberries was significantly reduced by freeze-drying in any operating condition, while the total polyphenol content was apparently augmented when a vacuum was used (compared to atmospheric pressure), an increase attributed to an improvement in the extractability of polyphenols. To conclude, for vitamin C and phenolic content retention, vacuum freeze drying most of the time gives the best results.

In terms of preserving β-carotene, lycopene, vitamin E, unsaturated oils, and other lipid-based oxidizable bio-compounds in fruits and vegetables, freeze-drying and storage of freeze-dried products should be taken with high consideration since autocatalytic oxidative reactions are accelerated at very low water activities achieved during freeze drying. As an example, Gutierrez et al. [45] indicated that oils from freeze-dried pulps of seabuckthorn berries had a much lower peroxide value than those obtained from air-dried berries, showing that low water activities attained during freeze-drying could damage the quality of lipid-based biocompounds.

## 6. Pretreatments and Process Intensification

Process intensification with innovative technologies (external to the product) or additional pretreatments (internal) prior to or during freeze-drying are key approaches aimed at efficiently overcoming processing challenges to increase mass transfer or improve product quality.

Chemical, mechanical, and thermal pretreatments have been used to reduce the effect of plant skin hydrophobicity and promote water transport during drying of whole berries. Chemical pretreatment involves immersion of the product in alkaline or acid solutions of oleate esters prior to drying. Alkaline dipping facilitates drying by forming cracks on the fruit surface [74]. However, the high temperature (100 °C) of the chemical solution and the long periods of soaking causes texture degradation and a low level of taste acceptability due to the incorporation of chemical residues in the fruit flesh [75]. Mechanical pretreatments might replace or complement chemical pretreatments, mainly because of the higher acceptability levels [76]. It consists of peeling, abrasion of the surface, puncturing the skin, or cutting the fruit in various shapes [77]. Araya-Farias et al. [44] halved sea-buckthorn berries before freeze-drying in order to produce high quality powders out of this oily, impermeable skinned fruit. Some other pretreatments include exposure to sulphur dioxide and thermal pretreatments such as blanching (immersion in hot water) or steaming [76]. However, blanching may cause the loss of soluble substances like proteins and mineral elements while high temperatures may induce the loss of heat labile substances such as nutrients and vitamins [78].

On the other hand, there has been little research done on freezing pretreatments prior to dehydration methods. Slow freezing helps the formation of large extracellular ice crystals damaging vegetable tissues while rapid freezing promotes intensive nucleation and formation of intracellular small ice crystals [79] and freeze-fractures and cracking in food tissues [80,81,82]. Water permeability of plant tissues depends on their composition [83], microstructure [84], crystalline or amorphous state of the matrix, and the lipid and glass transitions occurred during cooling or heating the tissues [85,86]. All these reported effects of freezing on vegetable and fruit tissues can certainly be used to induce positive changes in the food microstructure so as to increase drying rates or to improve dried product quality. Individual quick freezing (IQF), a rapid individual freezing of berries in a thin layer at −40 °C for a specified time [87], has been used in cycles with slow thawing in the refrigerator at 4 °C. This mild heat shock (−40 °C to +4 °C), together with the repetition in cycles, led to slight changes in the permeability of the waxy cuticle, sufficient to increase the drying rate [88].

Liquid nitrogen cyclic immersions of blueberries, seabuckthorn berries, and grapes markedly increased the drying kinetics during hot-air, vacuum, and freeze-drying [89]. The initial fruit epidermis thickness decreased between 20% to 50% (depending on the fruit) after 3–5 immersions in liquid N_2_. Also, dewaxing of the plant surface was observed after immersions in liquid nitrogen for lowbush (200.33 ± 3.05 to 152.70 ± 0.7 μg/cm^2^) and highbush (227.5 ± 2.12 to 112.17 ± 1.66 μg/cm^2^) blueberry cuticles [32], which explains the significant impact of this pretreatment on mass transfer acceleration during drying.

Dilution of a concentrated product is an easy solution to overcome problems indicated previously when dealing with liquid foods in high sugar/lipid concentrations. However, adding water and afterwards having to taking it out by an expensive method such as freeze-drying is not always an affordable solution unless necessary. Bhatta et al. [67] diluted maple syrup to 20% prior to freeze-drying as a first step of producing ultimate quality maple syrup powders. A pretreatment option for solving quality problems when freeze-drying high concentrated liquids, or solid plant foods having a moisture barrier (i.e., berries), is to provide the freeze-drier with frozen particulate systems instead of a tray of frozen liquid in a block. To achieve this, one simple way is to grind the frozen liquid at ultralow low temperatures. Then, the smaller size frozen particles are freeze-dried. This method was first reported for obtaining freeze-dried powders from plant tissues for botanical analytical use, by grinding the samples under liquid nitrogen, followed by freeze-drying [90]. One of the drawbacks, though, is a wide particle size distribution of the freeze-dried powder.

**Figure 5:**
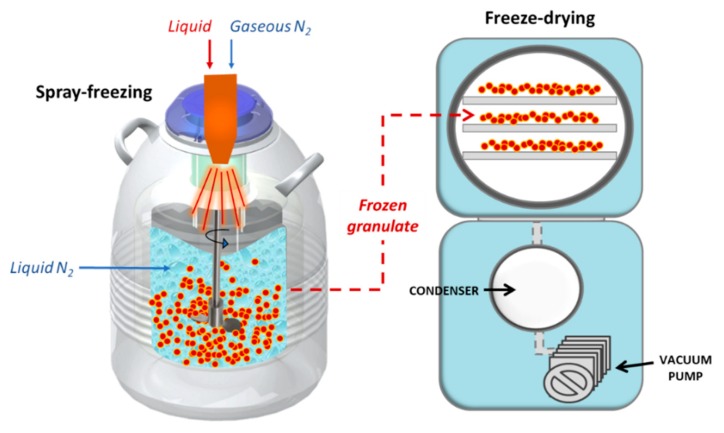
Freeze-granulation process.

The more recent freeze granulation technology involves spraying droplets of a liquid slurry or suspension into liquid nitrogen followed by freeze-drying of the frozen droplets [91]. The above-mentioned process is illustrated in Figure 5 (adapted from [92]). The significance of this technology is that the structure and homogeneity of the particles in the slurry or suspension are retained in the granules.

Another way of pretreating hard-to-freeze-dry solutions/suspensions/emulsions is by foaming, in the so-called ‘foam-mat freeze-drying’ process. In general, drying of foamed materials is faster than that of nonfoamed ones. Drying experts have repeatedly pointed to the increased interfacial area of foamed materials as the factor responsible for reduced drying time. However, because density of foamed materials is lower than that of nonfoamed ones and extends from 0.3 to 0.6 g/cm^3^, the mass load of the foam-mat dryer is also lower. Thus, shorter drying times should not only offset the reduced dryer load but also increase the dryer throughput. Raharitsifa & Ratti [93] revealed that freeze-drying of foamed apple juice was limited by heat transfer, while for nonfoamed one, by mass transfer. In this study, it was shown that the insulation property characteristic of foams was more significant in slowing down the freeze-drying process than the increased surface area available for mass transfer due to foaming. Although freeze-drying rates were increased by foaming, no practical minimal sample thickness could be found in order to increase freeze-dryer throughput as well. In further experiments, Raharitsifa & Ratti [94] reported that, confirming the glass transition temperature results, at 20 °C storage temperature and in presence of air, nonfoamed freeze-dried apple juice powders collapsed with marked change of color, while foamed freeze-dried products were stable for up to 70 days.

Some technical challenges facing freeze-drying include long residence times, batch operation mode, high operating cost, and energy consumption. It has been already pointed out that any new development to the classical vacuum freeze-drying should aim to improve heat transfer in order to help sublimation and reduce drying times, or to reduce/avoid the use of vacuum so as to decrease costs [3]. In recent years, studies have focused on development of process intensification technologies to resolve some of these issues.

Infrared energy impinges into the exposed material surfaces and propagates through the material to increase thermal energy through molecular vibration, which has relatively lower losses compared to other types of heat sources [95]. Application of infrared radiation in freeze-drying of plant-based foods significantly diminished drying time for sweet potato [96] and apple [97], and enhanced quality characteristics of the final product due to uniform surface heating [98], such as in the case of aloe vera [95], strawberry [99], and banana [100].

Lately, the application of microwave energy to intensify freeze-drying regained attention. Microwave heating has been studied since the 1970s in relation to the acceleration of freeze-drying [3]. The attractive aspect of this heating source is that it is an energy input that not only is essentially unaffected by the dry layers of the material undergoing freeze-drying, but also that is absorbed mainly in the frozen region [101]. Since the frozen region has a high thermal conductivity, microwave energy helps sublimation to decrease freeze-drying times up to 60–75% [102,103]. In addition, when compared to conventional freeze-drying, microwave assisted freeze-drying (MFD) may lead to products of similar/better quality [102,104,105]. Although microwave freeze-drying can offer unique advantages, the inherent problem preventing its commercialization is the difficulty in controlling the final product quality and assuring its uniformity, resulting from corona discharge and nonuniform heating, which cause ice melting and overheating [15]. In an interesting review of microwave assisted freeze-drying of foods, Duan et al. [104] pointed out that to assure a successful implementation of this type of technology in industry, the following challenges should be tackled: operation scale-up, accurate temperature monitoring, appropriate simulation of the MW field distribution, and increase in the knowledge on dielectric properties of foods. Thus, most of the numerous articles published lately considered the impact of microwave freeze-drying on different aspects of the final product quality and uniformity, such as the case for banana [106,107,108,109], barley grass [110], potato [108,111,112], mushrooms [113,114,115], apple [116,117,118], lettuce stem [65,119,120], okra [121], etc. In a recent review, [122] described an overview of the current developments in microwave assisted freeze-drying of fruits and vegetables, where they concluded the need for other novel nonthermal technologies, such as ultrasounds, high pressure processing, or pulsed electric fields, to improve quality of freeze-dried heat-sensitive fruits and vegetables.

Atmospheric freeze-drying (AFD) also has been getting increased attention recently. In this technique, freeze-drying is done at atmospheric pressure under inert dry gases. Although discovered at the end of the 1950s [123], this freeze-drying process started to pick up scientific interest mainly in the mid 1980s. Due to the lack of vacuum use, the cut off was approximately 34% as compared to vacuum freeze-drying [124]. However, drying times increased 1 to 3 times since the use of atmospheric pressure turns the control of the process from heat to mass transfer, which makes the kinetic extremely slow [3]. Other studies showed that in addition, quality of products was not excellent when atmospheric pressure is used instead of vacuum, since the risk of product collapse increased [125]. In order to accelerate drying kinetics, as well as improve quality issues, AFD has been combined with other techniques such as fluid-bed and spray freeze dryers [123]. As pointed out in this AFD review done by Claussen et al. [123], newer investigations of atmospheric freeze-drying in a fluid bed have looked into a process where a heat pump system is included with the drying system. 

More recently, power ultrasound proved to be an effective, nontoxic, and environmentally friendly way to speed the AFD process [126]. Repeated compression–expansion cycles generated by the ultrasound helps to create of micropathways in the solid to ease the vapor flow and microstirring at the-solid fluid interface, reducing the external mass transfer resistance [127]. Moreover, the additional exertion of a mild heating effect increased the interest in the ultrasound-assisted atmospheric freeze-drying of thermally sensitive products.

A compilation of the last 15 years of scientific publications in the area of atmospheric freeze-drying of plant-based foods can be found in Table 3, where their main objectives and conclusions are detailed. Most of these articles deal with improvements of the AFD process by the use of heat pump application to enhance economy aspects or final product quality, or by spout and fluidized beds with and without immersion adsorbents, pulverization by spray, set-up temperature programs, and ultrasound applications as drying strategies to accelerate AFD. Theoretical mathematical modeling [128,129,130] is an interesting strategy to understand the AFD process and simulate it under diverse conditions, to tackle AFD concrete problems on a solid basis. To end, it was surprising to see that little attention has been paid in some published works to verify that actual freeze-drying instead of air-drying of a frozen product was happening through all the process under atmospheric pressure. Compulsory continuous process humidity determinations and control should be included in future atmospheric freeze-drying studies.

## 7. Conclusions

Freeze-drying is widely used to dehydrate the plant-based foods including fruits, vegetables, spices, and even some nontraditional foods. Despite the long processing time and being an expensive drying method, it is preferred for the high final quality. Although some losses in vitamins and other valuable biocompounds can be found after freeze-drying, this type of dehydration method is the best to preserve nutritional qualities when compared to other dehydration methods, especially when operated under vacuum. In addition, quality parameters such as rehydration and porosity of freeze-dried foods are favorable for manufacturing variety of foods such as soup, instant drinks, cakes, etc. More recently, the process intensification of FD with innovative technologies or pretreatments allows overcoming some of the FD processing challenges.

## Figures and Tables

**Table 1:** Juice carbohydrate composition and glass transition temperatures.

Compound	Composition (% Total Carbohydrates)		*Tg* (°C) [40]
	Apple Juice (Gala Royal)	Pear Juice (Bartlett)	
*Sucrose*	11.7	4.7	52
*Glucose*	18.6	29.7	31
*Fructose*	65.4	49.6	100
*D-Sorbitol*	4.3	16.0	−2

**Table 2:** Some examples of freeze-dried fruits and vegetables.

Food [Reference]	Sample Preparation	FD Conditions	Key Quality Studied
Acai [54]	n/a	n/a	Antioxidant activity
Asparagus [13]	2–4 mm slices	T (shelf) = 20 °C T (condenser) = –64 °C Pressure = 3.3 kPa (3300 Pa) Time= 18–24 h	Rehydration; color; antioxidant; ascorbic acid
Blackberries [10]	Juice with carrier agents	T (not reported if shelf or condenser) = −84 °C Pressure = 0.0004 Pa Time = 48 h	Moisture; Thermal property; density; morphology; antiradical activity
Carrot [55]	3–4 mm slices	T (shelf) = 30 °C T (condenser) = –60 °C Pressure = 6 Pa Time = n/a	Moisture content; carotenoid content; lycopene content
Chinese gooseberry [48]	3 mm in height and 4 mm in diameter	2-step FD protocol (T shelf, time) = –20 °C for 20 h and +20 °C for 5 h Pressure = 10 Pa	Moisture content; Thermal properties; Sorption isotherm
Date [47]	Date pulp with carrier agents	T (not reported if shelf or condenser) = −40 °C Pressure = 42 Pa Time = 72 h	Moisture; powder flowability; morphology; microstructure
Guava and papaya [12]	1 cm cubes	T (shelf) = 10 °C Pressure = less than 613.2 Pa Time = 24 h	Color; porosity; rehydration; texture; Vitamin C
Green Pepper [49]	2 cm × 2 cm	T (not reported if shelf or condenser) = –47 to −50 °C Pressure = 0.666 Pa Time = 38 h	Texture; color
Pumpkin [49]	2 cm × 2 cm	T (not reported if shelf or condenser) = –47 to –50 °C Pressure = 0.666 Pa Time = 38 h	Texture; color
Pumpkin [6]	10 mm cubes	T (shelf) = 10 °C Pressure = 63 Pa Time = 24 h	Moisture content; water activity; color
Seabuckthorn berries [44]	Pulp and seeds	T (shelf) = 20 or 50 °C Pressure = 4 Pa Time = 24 h	Drying kinetics; nutritional composition
Seabuckthorn berries/leaves/seeds [56]	Crushed	FD Process conditions = n/a Time = 48 h	Moisture; water and oil absorption; color; structure; antiradical activity
Strawberries [14]	Sliced or whole fruits	T (shelf) = 30–70 °C Pressure = 4 Pa Time= 12, 24, or 48 h	Color;volume;collapse
Strawberries [46]	Half-cut or Sliced	T (shelf) = 55 °C Pressure = 4 Pa Time = 28 h	Rehydration; color; firmness
Tomatoes/Ginger [11]	Sliced	T (not reported if shelf or condenser) = −50 °C Pressure = 0.001330 Pa Time = 24 h	Total phenolic; ascorbic acid; antioxidant capacity
Tropical fruits (pineapple, Barbados cherry, guava, papaya, and mango) [57]	125 mm in diameter and 5 mm in height	T (not clear which temperature it is) = −30 °C Pressure = 0.001330 Pa Time = 12 h	Densities; porosity; nutritional property

n/a, not available.

**Table 3:** Publications on atmospheric freeze-drying of plant-based foods in the last 15 years (in chronological order of publication), where the following abbreviations are used: AFD = atmospheric freeze-drying, CDF = computational fluid mechanics, IAM = immersion in adsorbent material, URIF = uniformly retreat ice front.

Plant-Based Food	Objectives	Conclusions
Apple cubes [131]	To design and build a heat pump-assisted, packed bed AFD closed system and investigate the drying kinetics effect on the quality (rehydration kinetics, shrinkage, color, and antioxidant activity) of apple cubes	Mass diffusion controls the AFD process of apple dewatering at air temperatures below 0 °C. Process temperature had a major impact on final quality. The quality evaluation of apple cubes shows that dried products of AFD at −10 °C have similar rehydration kinetics and hygroscopic properties as the product obtained from vacuum freeze-drying.
Apple cubes [128]	To illustrate the construction and validation process of a CFD model at process temperatures below 0 °C.	CFD results based on film sublimation showed the viability of applying a surface sublimation model to AFD process. CFD results for apple cubes showed a predomination of inertial resistance of porous tissue. True values of tortuosity and internal resistance coefficient are critical for proper process simulation.
Apple cubes [132]	To test a robust and easy modeling tool for predicting AFD performance, designing and scaling-up of the AFD process where shrinkage is taken into consideration, and predictions of AFD drying kinetics can be performed at varying process temperature.	The diffusion model uses an effective diffusivity and activation energy to cover the AFD multicomponent diffusion mechanism. Coupled to shrinkage, the model showed good prediction of the drying kinetics of selected food products in the AFD process. Based on the predictions, it can be concluded that the diffusion model is capable of being applied to simulate AFD process for selected materials at constant and ascending process temperature modes.
Carrot slices [129]	To develop a mathematical model by adopting a sublimation–condensation model for the first stage of freeze-drying, solving the set of equations by fixed-finite-differences. Numerical simulations were carried out to analyze the characteristics of AFD in a fluidized bed dryer.	The complex interface movement in food products was well represented by the method of finite differences, using variable time steps that allowed significant reductions in computer time. The effect of particle size reduction, bed temperature increase, and the incorporation of infrared radiation made it possible to reduce primary drying times. The proposed model of AFD with one-directional mass and energy transport compared well to experimental data.
Carrot parallelepipeds [133]	To study the influence of particle size, freezing rate, air temperature, and mode of energy supply on both the final moisture content and particle shrinkage during AFD in a pulsed fluidized bed.	The air temperature was found to be the most important factor that affected the moisture content, followed by particle size, freezing rate, and type of energy supply. The air temperature was the only factor that affected shrinkage in AFD.
Peas [134]	To study the influence of drying temperatures and ultrasonic intensity on the effective acceleration of AFD rates.	Airborne ultrasound has high potential for improving AFD, as well as other processes that are based on heat and mass transfer rates at low temperatures.
Peas, apple and pinneaple cubes [135]	To use the Weibull model to represent AFD kinetics for different drying temperatures, drying times, approach velocities, products, and particle sizes.	The drying curves for several products obtained using this approach confirmed that AFD is controlled by internal diffusivity. The modified Weibull model adequately described the kinetics with high accuracy and enhanced stability.
Peas [136]	To investigate the application of microwave in AFD of green peas in a porous packed bed and its impact on drying kinetics and product quality.	Drying time was approximately halved when applying microwave radiation of 280 Watt into the process. Process temperature played a major role in product final quality with the lowest temperature being the most favorable to retain color.
Apple cubes [137]	To investigate the influence of different drying strategies on the AFD of apples.	A step-up temperature program based on glass transition temperature during AFD process can reduce the drying time by almost half on the premise of keeping product quality.
Apple cubes [130]	To evaluate the feasibility of a simple one-dimensional model to describe the ultrasonic assisted AFD process, as well as to validate such a model in different operating conditions.	A simple one-dimensional model was successfully applied to assess the effect of the ultrasonic application on the AFD kinetics under different conditions. US application is the parameter with the greatest influence on the AFD time and, consequently, is the key factor for the further optimization of the process.
Eggplant cubes [138]	To analyze the ultrasound-assisted AFD process and provide an in silico approach to the industrial process optimization. The URIF model was used to establish the kinetic parameters of the process.	Power ultrasound application significantly reduced the drying time. Air temperature and size of the samples also had a significant impact. The drying kinetics were successfully described using the URIF model. Power ultrasound can also increase the productivity of a tunnel dryer up to four or five times at industrial scale. Despite the benefits that can be envisioned by simulation, some limitations lie on the practice.
Wheat bran and vegetable pieces [139]	To characterize the hydrodynamic behavior of nonfood wheat bran, as potential adsorbent for AFD-IAM in a fluidized bed as well as spout-fluid bed and to study the segregation of binary mixtures of nonfood wheat bran and vegetables at different levels of dryness so as to establish the ideal conditions under which AFD-IAM can be performed without excessively reducing the product size.	Nonfood wheat bran is a promising material to be used as adsorbent. However, because it can be considered a “pseudo-cohesive” powder, potential difficulties in handling the binary mixture may occur when using a fluidized bed in the AFD process. Product density plays a fundamental role in mixing since poor contact between adsorbent and food material was found in the first stages of the AFD process (fluidized bed). Passive and active particle transport mechanisms and blocking effects of floor and roof were proposed to explain the observed behavior together with channeling and collapse cycle, allowing an explanation of the segregation phenomenon in fluidized beds and the mixing process in spout-fluid beds.
*Baccharis dracunculifolia* D.C. (Bd) plant extract [140]	To verify the influence of fluidized bed atmospheric spray-freeze-drying on the quality of Bd extracts as well as the physical and chemical stability of their main active species during and after the drying.	The main prenylated compounds of Bd are amenable to drying at freezing temperatures, d-mannitol showed an excellent cryoprotectant effect, decreasing the loss of all markers. Also, different powders obtained in the fluidized bed atmospheric spray-freeze-dryer showed adequate morphology, moisture, and excellent pharmaco-technical properties with good process yields. Fluid bed atmospheric spray-freeze-drying is an attractive alternative for processing heat-sensitive and high value-added crop products.
Eggplant cubes [127]	To evaluate the effect of air temperature and velocity, US power, and sample size on vitamin C, total phenolic, and antioxidant capacity of eggplant during US-assisted AFD	Power ultrasound is a promising technology for accelerating the AFD process, but attention must be paid to the optimization of the operating conditions in order to limit the thermal effects of acoustic energy and to ensure the preservation of the nutritional properties of the samples.
Mushroom slices [141]	To evaluate the feasibility of using power ultrasound to improve the AFD of mushroom, considering the kinetic effects and its influence on quality attributes.	Ultrasound represents an interesting means of significantly increasing the drying rate without producing important effects on the final quality of mushrooms.
